# The Effects of Catheter Ablation Therapy on Medication Use and Expenditures in Patients with Atrial Fibrillation

**DOI:** 10.36469/9881

**Published:** 2014-10-01

**Authors:** Matthew R. Reynolds, Guy David, Candace Gunnarsson, Jamie L. March, Steven C. Hao

**Affiliations:** 1 Lahey Clinic Medical Center, Burlington, MA; 2 University of Pennsylvania, Philadelphia, PA; 3 S2 Statistical Solutions, Inc., Cincinnati, OH; 4 Biosense Webster, Inc., Diamond Bar, CA; 5 Sutter Pacific Medical Foundation, San Francisco, CA

**Keywords:** atrial fibrillation, antiarrhythmic drugs, catheter ablation, medication expenditure, retrospective database analysis

## Abstract

**Background:** Atrial fibrillation (AF) is the most common cardiac arrhythmia encountered in clinical practice. Catheter ablation has become an important treatment option for many AF patients. Catheter ablation has been hypothesized to reduce the need for continued medical therapy for patients with AF, but there are few empirical data which demonstrate this.

**Objective:** The objective of this study was to estimate the impact of catheter ablation on antiarrhythmic drug (AAD) utilization and total drug expenditures among AF patients.

**Methods:** A retrospective analysis using the Truven Health Analytics MarketScan® Research Database was performed. Patients with AF and a catheter ablation procedure who had continuous enrollment in the database 6 months prior to their first ablation and a minimum of 1-year follow-up post first ablation were compared to AF patients who were treated with AADs and not ablation. Propensity matching was used to account for baseline differences between groups, and multivariable regression models adjusted for patient characteristics and baseline healthcare resource utilization. Sub-analyses were performed for patients age ≥65.

**Results:** AF patients treated with catheter ablation had significantly lower AAD utilization and total prescription drug costs than those treated with AADs only. These results persisted for the subset of patients age ≥65. The effects were strongest in the matched sample, where approximately 30% of ablation patients discontinued use of rhythm medication after receiving catheter ablation. Per-patient total medication expenditures were reduced by $800 to $1,200 per year in the matched sample.

**Conclusion:** Catheter ablation for AF reduced AAD utilization and total prescription drug expenditures in a sustainable fashion up to 3 years post ablation. This reduction was consistent and significant in both the non-Medicare and Medicare populations.

### BACKGROUND

Atrial fibrillation (AF) is the most common cardiac arrhythmia encountered in clinical practice. The prevalence of AF, which increases with age, is estimated at 0.4% of the general population.[1] In addition to reducing health-related quality of life[2] and exposing patients to increased risks of heart failure and stroke,[3,4] AF is also an expensive condition to treat, with recent studies projecting AF treatments costs in the United States as high as $10-$20 billion annually.[5,6]

Catheter ablation has become an important treatment option for many AF patients. Demonstrated benefits of AF ablation, relative to antiarrhythmic drug (AAD) therapy include superior maintenance of sinus rhythm,[7,8] improved quality of life[9] and possibly reduced hospitalization.[10]

Several published health economic analyses comparing ablation to AAD therapy have postulated that ablation, by reducing arrhythmia recurrence, may decrease long-term resource utilization and overall AF treatment costs.[11-13] Empirical evidence supporting this hypothesis is limited, particularly from general clinical practice. One previous study documented a reduction in drug expenditures following catheter ablation, but did not include a reference group not receiving ablation.[14]

The aim of this study was to compare AAD drug utilization and costs longitudinally in AF patients treated with catheter ablation versus those treated with AAD drugs but no ablation. This was done using a large, national claims database. The hypothesis was that patients treated with ablation would have reduced drug utilization and costs over time relative to those treated without ablation. In order to assess the potential impact of catheter ablation and drug expenditures specifically in Medicare-eligible patients, the analysis was stratified at an age cutoff of 65 years.

### METHODS

#### Data Source and Study Population

This study analyzed data from the U.S. MarketScan® Commercial Claims and Encounters Database and Medicare Supplemental Database from Truven Health Analytics℠ (New York, NY).[15] The databases are comprised of de-identified patient-level records from over 121 million patients since 1995 enrolled in employer-sponsored and public health insurance plans. In particular, they include data from 150 employers spanning several states. The MarketScan® Databases have been previously used for a variety of health services research studies and are in compliance with the Health Information Portability and Accountability Act of 1996. A protocol describing the study objectives, criteria for patient selection, data elements of interest, and statistical methods was submitted to the New England Institutional Review Board (NEIRB) and deemed exempt from review.

Patient-level data were extracted from the MarketScan Databases, covering the period between the first quarter of 2005 and the first quarter of 2009. Patients eligible for inclusion in the analysis were ≥18 years of age and had a primary diagnosis of AF (International Classification of Diseases, Ninth Revision, Clinical Modification [ICD-9] diagnosis code 427.31).[16] In addition, all patients were required to have 6 months of continuous medical and pharmacy enrollment in the database immediately prior to their respective baseline time and 1 year of continuous enrollment follow-up immediately afterwards.

Patients with AF were divided into ablation and AAD only groups using coding algorithms, as previously described.[17] Those in the ablation cohort were required to have an inpatient or outpatient visit with both a recorded diagnosis of AF and a documented catheter ablation procedure (ICD-9 code 37.34 or Current Procedural Terminology [CPT] code 93651). Patients with coexisting diagnoses for other supraventricular tachycardias or a procedure code for atrioventricular (AV) junction ablation, pacemaker or implantable cardioverter defibrillator insertion were excluded. For inpatient encounters, the ICD-9 codes for AF and catheter ablation were required to be designated as the primary diagnosis and procedure associated with the visit. A record of any cardiac ablation procedure in the six months prior to the procedure meeting the above requirements caused a patient to be excluded from the analysis. The analytic start time was defined for this cohort as the date of the first catheter ablation procedure meeting the inclusion/exclusion criteria.

Patients in the non-ablation cohort were also required to have a diagnosis of AF, but could not have any record of an ablation procedure. Additionally, they had to have documentation of at least two AAD prescription fills after their first recorded AF diagnosis. The first record of the second antiarrhythmic medication was then used as the analytic start time for this cohort.

Baseline patient characteristics were defined from available claims data in the 6 months prior to the analytic start time, including demographics, the presence or absence of comorbid conditions, history of medication usage for drug categories of interest, history of electrical cardioversion, and utilization of medical services. Demographics and other general descriptive variables included age, sex, procedure setting (inpatient/ outpatient), year of procedure, insurance and patient location variables, length of primary ablation hospital stay, and length of time in the database. The comorbid conditions of interest included various categories of structural heart disease, hypertension, diabetes, chronic obstructive pulmonary disease (COPD) and acute renal failure (Table 1).

#### Outcomes

Two outcomes of primary interest were defined: the likelihood of AAD usage, and overall prescription drug costs – including both insurer payments and patient co-payments – during pre-specified follow-up intervals. The reference period for this analysis was the 6 months prior to the first ablation procedure, or second AAD prescription (in patients not treated with ablation). AAD treatment was defined as a prescription fill for a Vaughan-Williams Class I or Class III antiarrhythmic drug[18] (i.e. propafenone, flecainide, sotalol, dofetilide, or amiodarone) at any time during a given 6-month interval. In order to compare follow-up results to the baseline period, these two outcomes were assessed in subsequent 6-month intervals, beginning with 6-12 months after the analytic start time. The interval from 0-6 months was not included, as this is often a period of medication titration or, in the case of ablation, repeat procedures and the intent of the study was to examine longer-term outcomes. This is analogous to a 6-month "blanking period", 3-month blanking is utilized in AF ablation clinical trials.[19, 20] Follow-up was truncated at 3 years, as few patients had continuous claims data available for longer than this.

#### Statistical Analysis

The primary objective was to compare longitudinal drug utilization and costs between AF patients who were and were not treated with ablation. Given that patient selection for catheter ablation in our study was non-random, significant baseline differences between the ablation and non-ablation patient groups were expected. Propensity matching was used to address these potential imbalances in baseline patient characteristics. Propensity scores for receipt of a catheter ablation were calculated for each of the patients included in the analysis based on a multivariable logistic regression model. This stepwise approach used 15 patient characteristics hypothesized to be associated with having a catheter ablation. These included: age group, gender, region of country, total enrollment in months, number of office visits, number of emergency department visits, rhythm medication usage (at baseline), warfarin usage, stroke or transient ischemic attack (TIA), COPD, heart failure, coronary artery disease, hypertension, cardiomyopathy and diabetes. Based on the propensity for having a cardiac catheter ablation procedure, ablation and non-ablation patients identified using our coding algorithm were matched with a four-digit nearest neighbor algorithm. The matching criteria were intentionally strict, knowing that a number of the ablation patients would not be found to have suitable non-ablation matches, in order to make the matched ablation and non-ablation cohorts as similar as possible.

Table 1. Clinical and Demographic Characteristics at the Baseline Period*

| | All Patients | | Patients Age ≥65 years | | All Patients (matched Sample) | |
|---|---|---|---|---|---|---|
| | **Ablation n = 2645** | **No Ablation n = 7162** | **Ablation n = 761** | **No Ablation n = 4672** | **Ablation n = 801** | **No Ablation n = 801** |
| **Demographics** | | | | | | |
| Mean age (SD) | 59.2 (10.7) | 69 (11.4) | 71.3 (5.1) | 75.9 (6.4) | 63.3 (10.4) | 63.6 (11.0) |
| Male | 72% | 55% | 60% | 49% | 61% | 62% |
| Female | 28% | 45% | 40% | 51% | 39% | 38% |
| **Region** | | | | | | |
| Northeast | 9% | 7% | 6% | 7% | 7% | 8% |
| South | 38% | 37% | 31% | 34% | 38% | 40% |
| West | 20% | 19% | 24% | 20% | 17% | 19% |
| North central | 33% | 37% | 38% | 39% | 37% | 33% |
| **Insurance Plan Characteristics** | | | | | | |
| Comprehensive | 22% | 41% | 51% | 56% | 30% | 32% |
| EPO | 1% | 0.20% | 0% | 0% | 0.20% | 0.40% |
| HMO | 13% | 10% | 6% | 6% | 11% | 12% |
| POS | 8% | 5% | 3% | 2% | 8% | 7% |
| PPO | 53% | 43% | 40% | 36% | 49% | 46% |
| POS capitated | 1% | 0.20% | 0% | 0% | 0.40% | 0.10% |
| CDHP or HDHP | 2% | 1% | 0.40% | 0.20% | 1% | 2% |
| **Clinical Characteristics** | | | | | | |
| Structural heart disease | | | | | | |
| Ischemic with MI | 1% | 4% | 2% | 5% | 2% | 2% |
| Heart failure | 10% | 23% | 14% | 27% | 17% | 16% |
| Cardiomyopathy | 7% | 9% | 4% | 9% | 7% | 7% |
| Hypertensive heart disease without heart failure | 3% | 4% | 3% | 4% | 3% | 4% |
| Valvular heart disease | 21% | 25% | 24% | 26% | 25% | 21% |
| Conduction system disease | 5% | 6% | 5% | 7% | 5% | 6% |
| Congenital heart disease | 3% | 2% | 2% | 2% | 3% | 2% |
| Hypertension | 36% | 45% | 40% | 46% | 43% | 41% |
| Diabetes | 13% | 17% | 15% | 18% | 19% | 15% |
| COPD | 6% | 14% | 11% | 17% | 10% | 11% |
| Acute renal failure | 1% | 3% | 2% | 4% | 2% | 2% |
| Use of rhythm medication | 66.24% | 83.20% | 63.34% | 81.80% | 77.40% | 77.65% |
| Drug expenditures (SD) | 1841 (1794) | 1936 (1888) | 2024 (1934) | 2016 (1838) | 1982 (1669) | 1815 (1761) |

Note: Clinical and demographic characteristics are described in the baseline period, 6 months pre-index. The first record of the second antiarrhythmic medication is defined by attrition rules as index or day 0 for the non-ablation group; CDHP: consumer driven health plan; COPD: chronic obstructive pulmonary disease; EPO: exclusive provider organization; HDHP: high deductible health plan; HMO: health maintenance organization; POS: point of service; PPO: preferred provider organization; MI: myocardial infarction; SD: standard deviation

### RESULTS

#### Population Characteristics

Using the coding algorithm described above, 2,645 patients were identified as meeting study entry criteria who underwent a first catheter ablation procedure for AF between 2005 and 2009 (Supplemental Figure 1). Of these patients, 761 were ≥ age 65. In addition, 7,162 AF patients were identified as treated with at least two AADs but not ablation (Supplemental Figure 2). Of these, 4,672 were age ≥65.

Baseline clinical and demographic characteristics of the ablation and non-ablation patients are summarized in Table 1. In the entire population, the majority of patients were male and insured by a preferred provider organization (PPO). Patients undergoing AF ablation compared with patients who did not receive the procedure were younger (mean 59.2 vs. 69.0 years) and were more often male (72% vs. 55%), and insured by a PPO (53% vs. 43%). The differences in age and gender persisted in the age ≥65 sample. Moreover, patients undergoing catheter ablation for AF were less likely to have certain comorbidities, such as heart failure, hypertension, COPD and diabetes. The lower panel of Table 1 reports summary statistics for the two outcome variables in the 6 months prior to the ablation procedure or second AAD prescription. As expected, the proportion of patients receiving AADs in the 6-month baseline period was higher in the ablation versus non-ablation groups. Despite this, total baseline drug expenditures were similar (<$200 difference) for both groups. After matching, age, gender, comorbidities and medication utilization were balanced between the ablation and medication cohort (Table 1).

#### Antiarrhythmic Drug Utilization

The frequency of AAD utilization over time for the ablation and non-ablation groups within the propensity matched sample (N=1,602) is shown in Figure 1A. In the 6 months prior to the analytic start time, just under 80% of patients in both groups had been treated with AADs. In the non-ablation group, AAD use fell gradually to just over 60% between 2.5 and 3 years. AAD use fell more quickly and remained near 40% after 12 months in the ablation group. Results were similar in the older subset of patients (Figure 1B), although AAD use in older patients not treated with ablation fell to a slightly greater extent.

Results of the regression models on the outcome of AAD use per 6-month interval are shown in Table 2. Results were similar across levels of multivariable adjustment. As indicated by the ablation by time interaction terms (top row of results in table), in the matched sample patients treated with ablation were 25-37% less likely to be treated with AADs during follow-up than patients not treated with ablation. Results were similar in the matched sample of patients age 65 and over, as shown in the lower panel of Table 2.

**Figure 1A.** Percentage of Patients in the Matched Sample Receiving Rhythm Medication (N=1,602 at baseline)

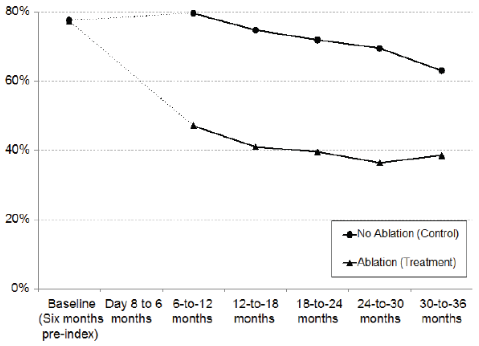

**Figure 1B.** Percentage of Patients in the Matched Sample over Age 65 Receiving Rhythm Medication (N=738)

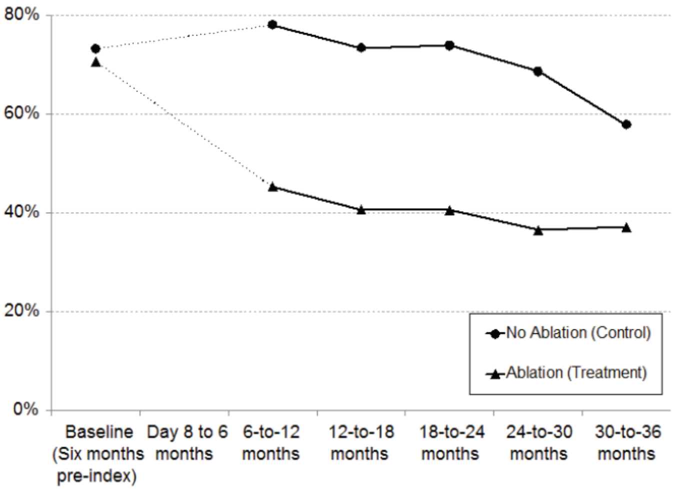

**Table 2.** Use of Rhythm Medication

| Matched Sample - All | 6 Months to 1 Year | | 1 year to 1.5 years | | 1.5 years to 2 years | | 2 years to 2.5 years | | 2.5 years to 3 years | |
|---|---|---|---|---|---|---|---|---|---|---|
| Number of Patients | 1602 | 1602 | 843 | 843 | 438 | 438 | 224 | 224 | 108 | 108 |
| Ablation x Post | -0.323* | -0.370* | -0.336* | -0.364* | -0.308* | -0.326* | -0.331* | -0.351* | -0.257* | -0.293* |
| | [0.0308] | [0.0316] | [0.0353] | [0.0359] | [0.0426] | [0.0436] | [0.0527] | [0.0542] | [0.0698] | [0.0719] |
| Ablation (treatment) | -0.0025 | -0.00579 | -0.00171 | -0.00953 | -0.0164 | -0.031 | | 0.0216 | 0.0116 | 0.00938 |
| | [0.0209] | [0.0211] | [0.0236] | [0.0244] | [0.0282] | [0.0295] | [0.0344] | [0.0387] | [0.0437] | [0.0536] |
| Post Period Dummy | 0.02 | 0.0365 | -0.0254 | 0.00872 | -0.0654** | -0.0481 | | -0.058 | -0.177* | -0.124** |
| | [0.0205] | [0.0228] | [0.0233] | [0.0258] | [0.0285] | [0.0323] | [0.0356] | [0.0404] | [0.0465] | [0.0535] |
| Constant | 0.777* | 1.054* | 0.773* | 1.006* | 0.784* | 0.908* | 0.792* | 0.836* | 0.807* | 1.066* |
| | [0.0147] | [0.0858] | [0.0162] | [0.111] | [0.0192] | [0.145] | [0.0236] | [0.229] | [0.0294] | [0.364] |
| R-squared | 0.088 | 0.129 | 0.104 | 0.146 | 0.11 | 0.147 | 0.133 | 0.175 | 0.128 | 0.196 |
| Controls | | § | | § | | § | | § | | § |
| **Matched Sample Age ≥65** | **6 Months to 1 Year** | | **1 year to 1.5 years** | | **1.5 years to 2 years** | | **2 years to 2.5 years** | | **2.5 years to 3 years** | |
| Number of Patients | 738 | 738 | 387 | 387 | 204 | 204 | 102 | 102 | 49 | 49 |
| Ablation x Post | -0.354* | -0.399* | -0.371* | -0.381* | -0.336* | -0.334* | -0.359* | -0.411* | -0.255** | -0.357* |
| | [0.0472] | [0.0484] | [0.0541] | [0.0549] | [0.0640] | [0.0656] | [0.0818] | [0.0840] | [0.109] | [0.116] |
| Ablation (treatment) | 0.0258 | 0.0158 | 0.0434 | 0.0152 | 0.00193 | -0.0353 | 0.038 | 0.0606 | 0.0477 | 0.0963 |
| | [0.0331] | [0.0342] | [0.0377] | [0.0393] | [0.0443] | [0.0474] | [0.0559] | [0.0670] | [0.0723] | [0.0986] |
| Post Period Dummy | 0.0739** | 0.112* | 0.0455 | 0.0857** | 0.0153 | 0.0466 | -0.0229 | 0.0435 | -0.145 | -0.0097 |
| | [0.0328] | [0.0374] | [0.0379] | [0.0428] | [0.0448] | [0.0523] | [0.0569] | [0.0674] | [0.0769] | [0.0938] |
| Constant | 0.707* | 1.475* | 0.689* | 1.612* | 0.724* | 1.654* | 0.710* | 1.602* | 0.724* | 0.969 |
| | [0.0243] | [0.212] | [0.0274] | [0.253] | [0.0320] | [0.319] | [0.0398] | [0.536] | [0.0516] | [0.888] |
| R-squared | 0.074 | 0.14 | 0.081 | 0.147 | 0.089 | 0.168 | 0.098 | 0.21 | 0.099 | 0.238 |
| Controls | | § | | § | | § | | § | | § |

*p<0.01; **p<0.05; Robust standard errors in brackets; The top panel shows results of multivariable regression models for rhythm medication utilization for the propensity matched cohort, the bottom panel shows results for patients over age 65. Results are displayed for five samples, based on duration of available follow-up time post ablation. For each post-ablation period, two sets of results are reported. §The first includes no controls (first column) and the second presents the most saturated model, which includes all clinical and demographic controls (second column). For patients matched on clinical dimensions at the baseline, the effect of ablation was stronger, with the likelihood of using rhythm medications falling approximately 30%.

**Figure 2A.** Difference in Drug Expenditures in the Matched Sample (N=1,602)

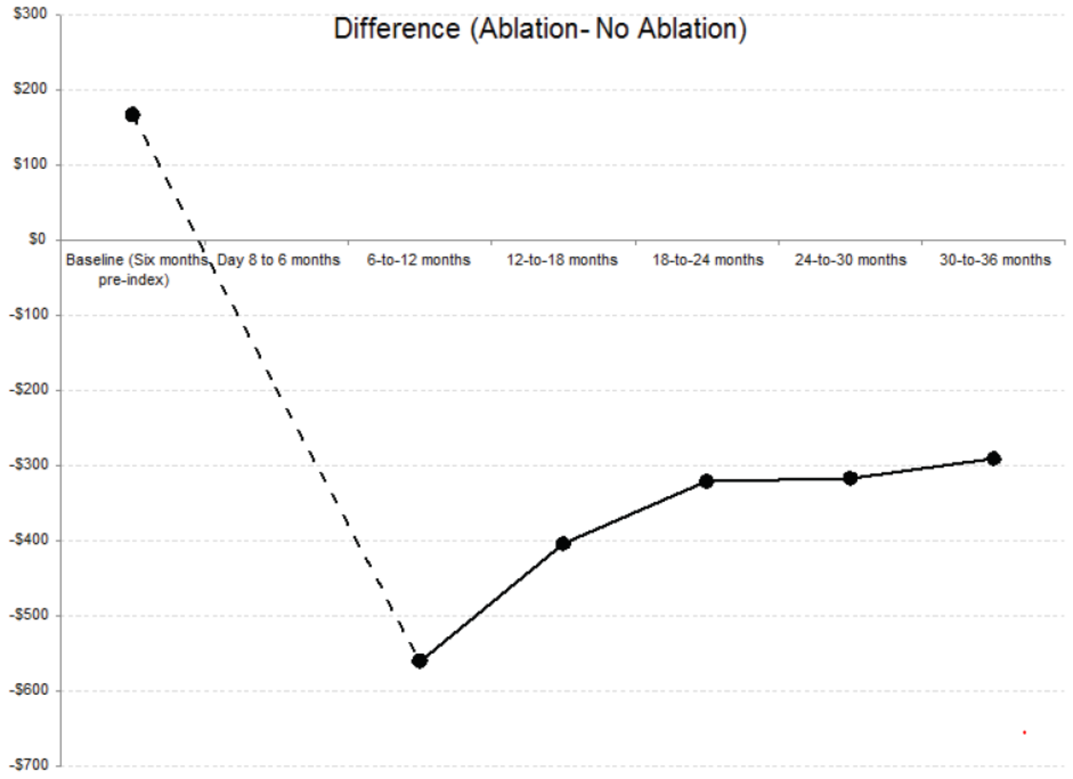

**Figure 2B.** Difference in Drug Expenditures in the Matched Sample Age ≥65 (N=738)

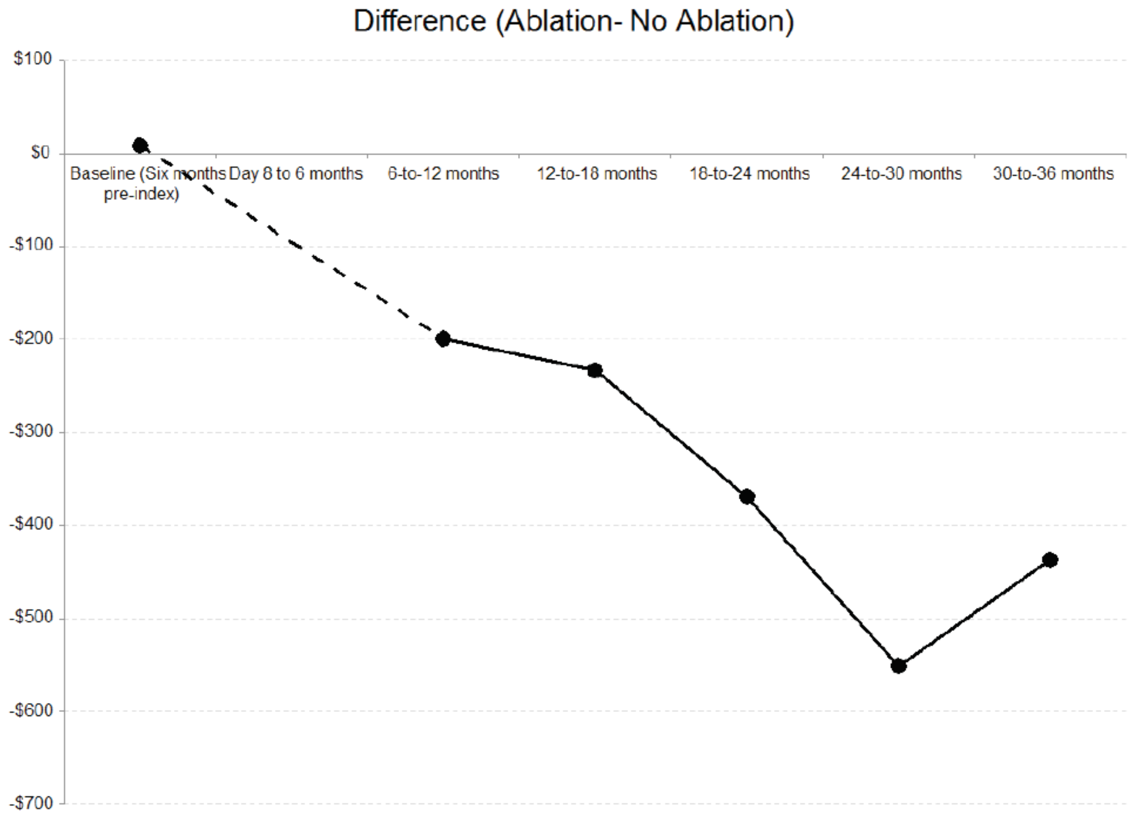

### Overall Prescription Drug Expenditures

Results of the regression models on the outcome of total prescription drug expenditures are shown in Table 3. The difference between the ablation group and non-ablation group in semi-annual drug costs is shown graphically in Figure 2A and in Figure 2B for the subset of patients aged ≥65 years. These differences were roughly $400-$600 (per 6 months) for the full matched sample, and $200-$400 for patients age ≥65.

**Table 3.** Expenditures on Medication – Matched Sample

| | 6 months to 1 year | | 1 to 1.5 years | | 1.5 to 2 years | | 2 to 2.5 years | | 2.5 to 3 years | |
|---|---|---|---|---|---|---|---|---|---|---|
| Number of Patients | 1365 | 1365 | 719 | 719 | 377 | 377 | 192 | 192 | 92 | 92 |
| **Ablation x Post** | -588.2* | -604.5* | -478.5* | -502.6* | -569.9* | -606.5* | -591.7** | -589.1* | -457.3 | -401.1 |
| | [163.2] | [153.2] | [174.4] | [163.5] | [207.2] | [203.7] | [235.8] | [224.4] | [301.4] | [298.9] |
| Ablation (treatment) | 237.6** | 181.9** | 118.2 | -0.318 | 207.2*** | 137.9 | 6.618 | 193.7 | 166.5 | 427.8* |
| | [92.88] | [91.99] | [102.4] | [108.3] | [109.9] | [121.2] | [142.2] | [166.5] | [189.3] | [220.9] |
| Post Period Dummy | 463.1* | 250.8*** | 290.3** | 122.2 | 421.8* | 134.6 | 266.1 | -34.49 | 198.8 | 6.92 |
| | [129.7] | [139.6] | [140.2] | [142.8] | [155.5] | [177.4] | [187.0] | [179.4] | [226.7] | [246.7] |
| Constant | 1817* | 1028** | 1865* | 734.4 | 1811* | 1222 | 1912* | 123.9 | 1815* | -1581 |
| | [67.48] | [446.4] | [79.04] | [592.8] | [76.85] | [806.5] | [105.3] | [944.9] | [132.5] | [1284] |
| R-squared | 0.006 | 0.144 | 0.004 | 0.137 | 0.007 | 0.153 | 0.012 | 0.178 | 0.004 | 0.174 |
| Controls | | § | | § | | § | | § | | § |

*p<0.01; **p<0.05, ***p<0.1; Robust standard errors in brackets; Results of multivariable regression models for drug expenditures for the propensity matched cohort. Results displayed for five samples, based on duration of available follow-up time post ablation. For each post-ablation period, two sets of results are reported. § The first includes no controls (first column) and the second presents the most saturated model, which includes all clinical and demographic controls (second column). In the matched sample, the effects of ablation were stronger, with drug expenditures falling between $800 and $1,200 per year (between $401 and $606 per 6 months).

### Discussion

This study found that catheter ablation in patients with AF was associated with a clinically significant and durable reduction in rhythm medication utilization, as compared with AAD treatment without ablation. These reductions were concomitant with declines in overall medication expenditures. The results were similar in a sub-analysis of patients who were age ≥65, although savings on total prescription drug expenditures in this population were slightly smaller.

These findings to some extent validate assumptions made in several previous health economic analyses of AF ablation regarding long-term medical therapy costs,[11-13] and have the advantage of being based on data from patients in general U.S. practice. The results are, therefore, likely to be generalizable to the patients currently being managed with AF ablation across the country.

From a clinical perspective, the continued use of AADs in patients undergoing AF ablation may be slightly higher than expected, but most likely not by a large margin. Both a previous meta-analysis of AF ablation studies and a voluntary worldwide survey on AF ablation found that the rate of repeat procedures after a first AF ablation is in the range of 25%,[21, 22] and randomized trials of AF ablation for paroxysmal AF have reported 12 month rates of freedom from atrial arrhythmia of 65-70%.[20,23]

Since the adjunctive use of AADs may improve the results of ablation,21 the observation that roughly 40% of patients continued AAD therapy may be a realistic representation of results in general clinical practice, though one not previously reported with up to 3 years of follow-up. These results are also consistent with prior literature reagarding AF patients not treated with catheter ablation, documenting relatively high rates of AAD discontinuation due to both adverse events and inefficacy.[24-26]

Few previous studies have documented the potential cost savings that may be associated with reductions in prescription drug utilization following AF ablation. Using a similar data source, Ladapo et al.[14] recently showed reductions in multiple categories of healthcare resource utilization, including prescription drug costs, after AF ablation, compared with prior to ablation, but that study did not include a comparison group of AF patients treated with AADs and not ablation. Shah and colleagues[27] also recently reported on outcomes after AF ablation using a California claims database, documenting a 30% rate of readmission for atrial arrhythmia or repeat ablation over 2 years, but their study did not examine prescription drug use or costs.

#### Limitations

These data should be interpreted with the following limitations in mind. First and most importantly, the clinical decision to perform catheter ablation on an AF patient is not a random one, as evidenced by the marked differences between the ablation and non-ablation patients in this study prior to propensity matching. It is clear that patients selected for ablation are younger and healthier than those not treated with ablation. Multiple techniques were used to adjust for these expected and observed differences between patient groups, including propensity matching and multivariable regression modeling. Only a large-scale randomized trial would be able to control for potential unmeasured confounders, however recent randomized trials in this field have been characterized by high rates of crossover from drugs to ablation, which would preclude the kinds of comparisons done in this study.

This study is also subject to the usual limitations of research done using claims data. The Medicare sample used in this analysis consists of patients that have Medicare supplemental insurance (e.g. Medicare Advantage plans), which, on average, include patients with slightly higher socioeconomic status compared to Medicare Fee-for-Service patients. Additionnally, during the period of the study, there was not a unique billing code for AF ablation, thus the case finding relied on a coding algorithm which was designed with high specificity by excluding patients who might have had procedures for arrhythmias other than AF.

In addition to potential inaccuracies inherent in claims data, this type of data source also lacks clinical detail. It was not possible to tell, for example, what the success rates of either AAD treatment or catheter ablation were in suppressing AF, or what the reasons were for discontinuing AADs in any particular patient. Since AAD use fell sharply (within 12 months) in the ablation group and more gradually (over 2-3 years) in the non-ablation group, it is tempting to suggest that the reasons for drug discontinuation may have differed between groups. Linkage with a richer clinical data source would be required to understand this.

### CONCLUSION

AF patients treated with catheter ablation are significantly more likely to discontinue AADs over a 2- to 3-year period than patients initially treated with AADs, but not ablation. This was associated with savings of as much as $800-$1,200 per year in overall prescription drug expenditures in some patient groups, but more modest reductions in older patients. These data support the view that the up-front costs of AF ablation are at least partially offset by decreased health resource utilization over time.

## APPENDIX

**Supplemental Figure 1.** Selection Algorithm for Patients with Catheter Ablation

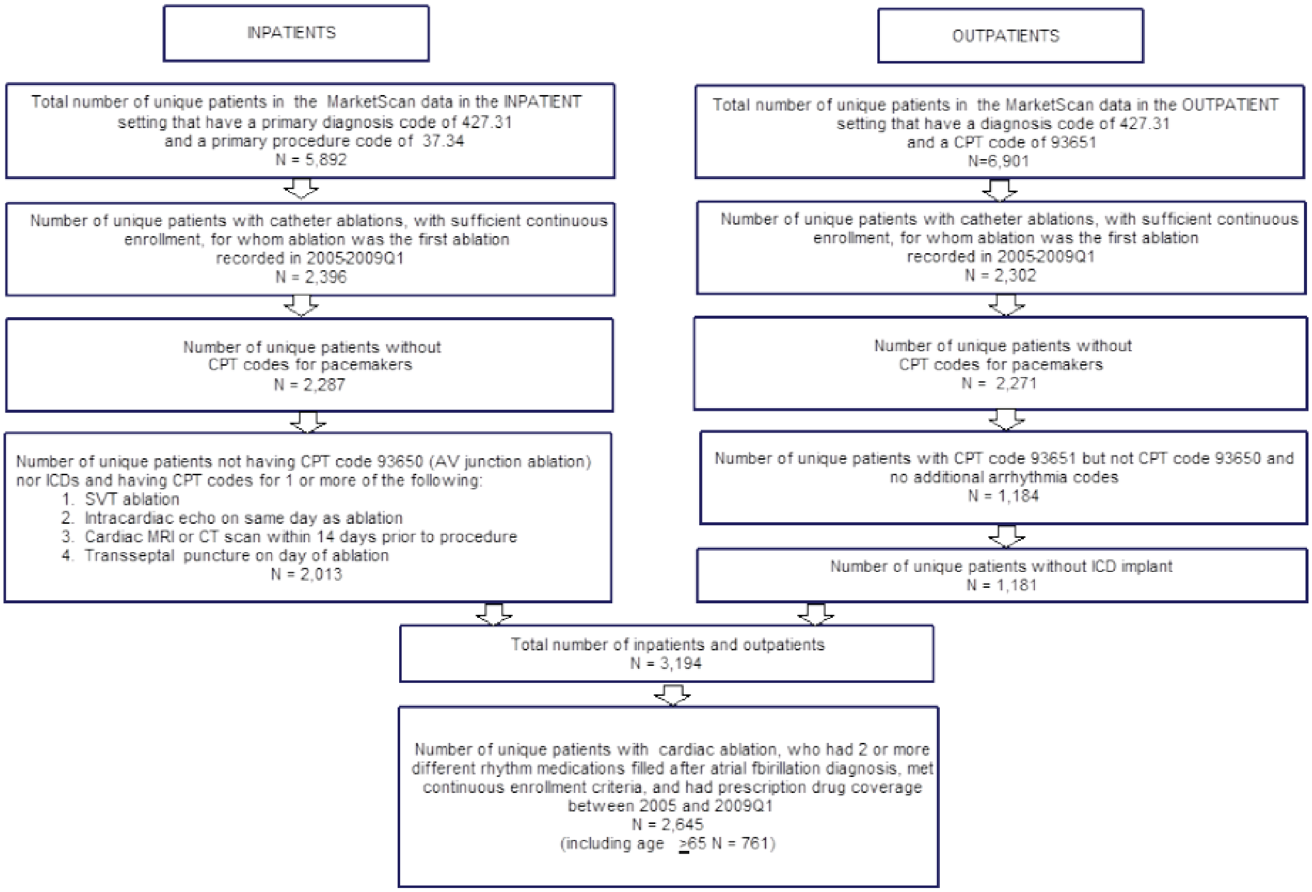

CPT: current procedural terminology; AV: atrioventricular; SVT: supraventricular tachycardia; MRI: magnetic resonance imaging; CT: computed tomography; ICD: implantable cardioverter-defibrillator

**Supplemental Figure 2.** Selection Algorithm for the Patients Without Catheter Ablation

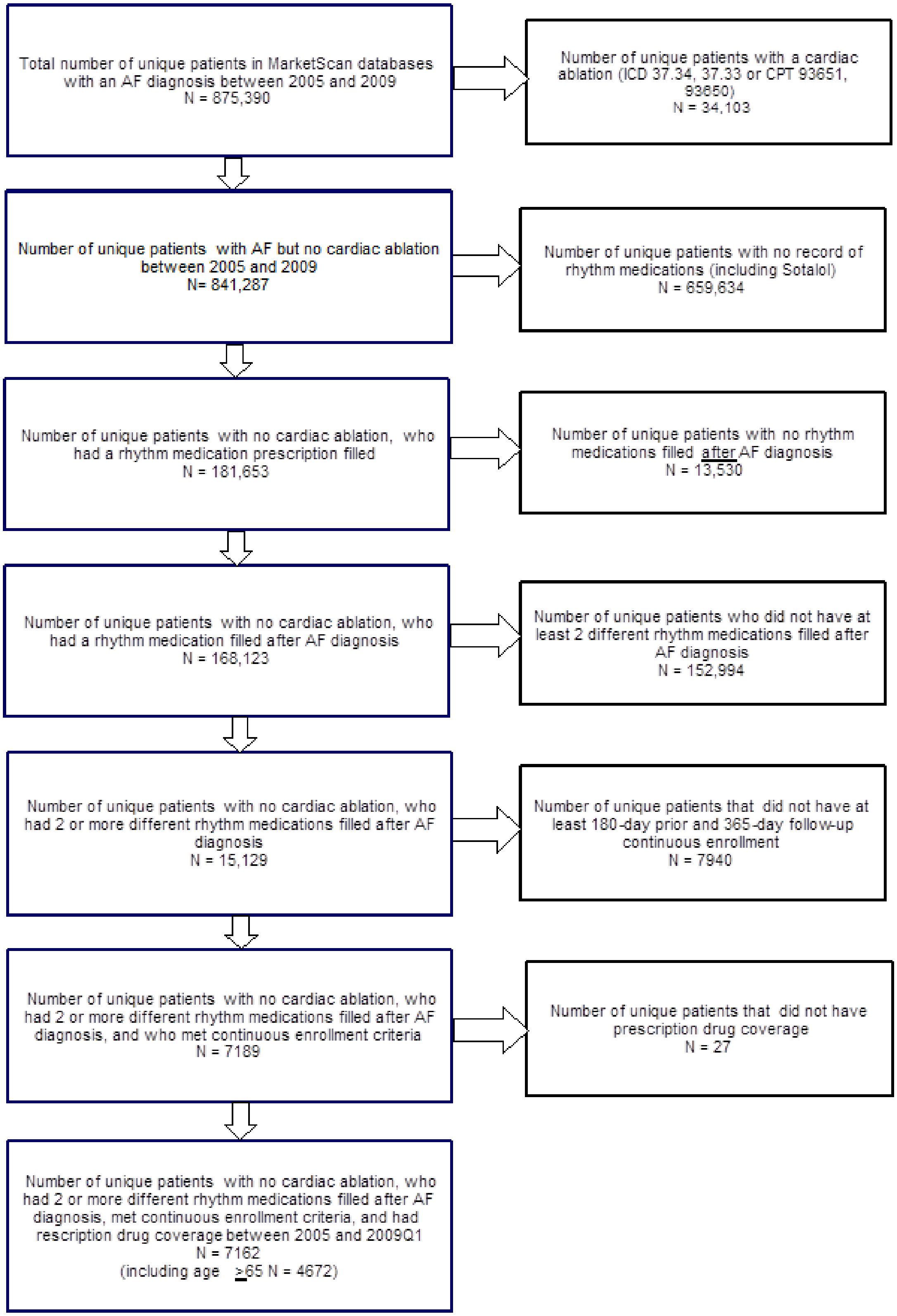

AF: Atrial Fibrillation

### CONFLICT OF INTEREST DECLARATION

This study was funded by Biosense Webster, Inc. Matthew Reynolds, MD, MSc is a consultant for Biosense Webster, Inc., and Medtronic. Guy David PhD is an academic affiliate to S2 Statistical Solutions, Inc. Candace Gunnarsson, EdD is an employee of S2 Statistical Solutions, Inc. which is a paid consultant for Biosense Webster, Inc. Jamie March, MBA, is an employee of Biosense Webster, Inc., a Johnson & Johnson Company. Steven Hao, MD, FACC, FHRS is on the advisory board and received consulting fees from Biosense Webster, Inc. and received honorarium (teaching/speaking) fees from St. Jude Medical and Medtronic. The publication of study results was not contingent on the sponsor's approval or censorship of the manuscript.

## References

[1] Furberg CD, Psaty BM, Manolio TA, et al: Prevalence of atrial fibrillation in elderly subjects (the Cardiovascular Health Study). *Am J Cardiol* 1994;74(3):236-41.

[2] Reynolds MR, Lavelle T, Essebag V, et al: Influence of age, sex, and atrial fibrillation recurrence on quality of life outcomes in a population of patients with new-onset atrial fibrillation: the Fibrillation Registry Assessing Costs, Therapies, Adverse events and Lifestyle (FRACTAL) study. *Am Heart J* 2006;152(6):1097-103.

[3] Dries DL, Exner DV, Gersh BJ, et al: Atrial fibrillation is associated with an increased risk for mortality and heart failure progression in patients with asymptomatic and symptomatic left ventricular systolic dysfunction: a retrospective analysis of the SOLVD trials. Studies of Left Ventricular Dysfunction. *J Am Coll Cardiol* 1998;32(3):695-703.

[4] Wolf PA, Abbott RD, Kannel WB: Atrial fibrillation as an independent risk factor for stroke: the Framingham study. *Stroke* 1991;22:983-8.

[5] Lee WC, Lamas GA, Balu S, et al: Direct treatment cost of atrial fibrillation in the elderly American population: a Medicare perspective. *J Med Econ* 2008;11(2):281-98.

[6] Kim MH, Lin J, Hussein M, et al: Cost of atrial fibrillation in United States managed care organizations. *Adv Ther* 2009;26(9):847-57.

[7] Cosedis Nielsen J, Johannessen A, Raatikainen P, et al: Radiofrequency ablation as initial therapy in paroxysmal atrial fibrillation. *N Engl J Med* 2012;367(17):1587-95.

[8] Ip S, Terasawa T, Balk EM, et al: Comparative effectiveness of radiofrequency catheter ablation for atrial fibrillation. Comparative Effectiveness Review No. 15. (Prepared by Tufts Medical Center Evidence-based Practice Center under Contract No. 290-02-0022) Rockville, MD: Agency for Healthcare Research and Quality. 2009 July.

[9] Reynolds MR, Walczak J, White SA, et al: Improvements in symptoms and quality of life in patients with paroxsymal atrial fibrillation treated with radiofrequency catheter ablation versus antiarrhythmic drugs. *Circ Cardiovasc Qual Outcomes* 2010;3(6):615-23.

[10] Piccini JP, Lopes RD, Kong MH, et al: Pulmonary vein isolation for the maintenance of sinus rhythm in patients with atrial fibrillation: a meta-analysis of randomized, controlled trials. *Circ Arrhythm Electrophysiol* 2009;2(6):626-33.

[11] Khaykin Y, Morillo CA, Skanes AC, et al: Cost comparison of catheter ablation and medical therapy in atrial fibrillation. *J Cardiovasc Electrophysiol* 2007;18:907-13.

[12] McKenna C, Palmer S, Rodgers M, et al: Cost-effectiveness of radiofrequency catheter ablation for the treatment of atrial fibrillation in the United Kingdom. *Heart* 2009;95(7):542-9.

[13] Reynolds MR, Zimetbaum P, Josephson ME, et al: Cost-effectiveness of radiofrequency catheter ablation compared with antiarrhythmic drug therapy for paroxysmal atrial fibrillation. *Circ Arrhythmia Electrophysiol* 2009;2:362-9.

[14] Ladapo JA, David G, Gunnarsson CL, et al: Healthcare utilization and expenditures in patients with atrial fibrillation treated with catheter ablation. *J Cardiovasc Electrophysiol* 2012;23(1):1-8.

[15] Thomson Reuters MarketScan Research Data 2009. http://marketscan.thomsonreuters.com/marketscanportal/. Accessed January 19, 2011.

[16] Buck CJ: 2011 ICD-9-CM for Physicians, Vol. 1 and 2. St. Louis, MO: Elsevier Saunders, American Medical Association; 2011.

[17] Reynolds MR, Gunnarsson CL, Hunter TD, et al: Health outcomes with catheter ablation or antiarrhythmic drug therapy in atrial fibrillation: results of a propensity-matched analysis. *Circ Cardiovasc Qual Outcomes* 2012;5(2):171-81.

[18] Vaughan Williams EM: A classification of antiarrhythmic actions reassessed after a decade of new drugs. *J Clin Pharmacol* 1984;24(4):129-47.

[19] Calkins H, Kuck KH, Cappato R, et al: 2012 HRS/EHRA/ECAS expert consensus statement on catheter and surgical ablation of atrial fibrillation: recommendations for patient selection, procedural techniques, patient management and follow-up, definitions, endpoints, and research trial design: a report of the Heart Rhythm Society (HRS) Task Force on Catheter and Surgical Ablation of Atrial Fibrillation. Developed in partnership with the European Heart Rhythm Association (EHRA), a registered branch of the European Society of Cardiology (ESC) and the European Cardiac Arrhythmia Society (ECAS); and in collaboration with the American College of Cardiology (ACC), American Heart Association (AHA), the Asia Pacific Heart Rhythm Society (APHRS), and the Society of Thoracic Surgeons (STS). Endorsed by the governing bodies of the American College of Cardiology Foundation, the American Heart Association, the European Cardiac Arrhythmia Society, the European Heart Rhythm Association, the Society of Thoracic Surgeons, the Asia Pacific Heart Rhythm Society, and the Heart Rhythm Society. *Heart Rhythm* 2012;9(4):632-96 e21.

[20] Wilber DJ, Pappone C, Neuzil P, et al: Comparison of antiarrhythmic drug therapy and radiofrequency catheter ablation in patients with paroxysmal atrial fibrillation: a randomized controlled trial. *JAMA* 2010;303(4):333-40.

[21] Calkins H, Reynolds MR, Spector P, et al: Treatment of atrial fibrillation with anti-arrhythmic drugs or radio frequency ablation: two systematic literature reviews and meta-analyses. *Circ Arrhythmia Electrophysiol* 2009;2:349-61.

[22] Cappato R, Calkins H, Chen SA, et al: Updated worldwide survey on the methods, efficacy, and safety of catheter ablation for human atrial fibrillation. *Circ Arrhythm Electrophysiol* 2010;3(1):32-8.

[23] Packer DL, Kowal RC, Wheelan KR, et al: Cryoballoon ablation of pulmonary veins for paroxysmal atrial fibrillation: first results of the North American Arctic Front (STOP AF) pivotal trial. *J Am Coll Cardiol* 2013;61(16):1713-23.

[24] Freemantle N, Lafuente-Lafuente C, Mitchell S, et al: Mixed treatment comparison of dronedarone, amiodarone, sotalol, flecainide, and propafenone, for the management of atrial fibrillation. *Europace* 2011;13(3):329-45.

[25] Zimetbaum P, Ho KKL, Olshansky B, et al: Variation in the utilization of antiarrhythmic drugs in patients with new-onset atrial fibrillation. *Am J Cardiol* 2003;91:81-3.

[26] Singh BN, Singh SN, Reda DJ, et al: Amiodarone versus sotalol for atrial fibrillation. *N Engl J Med* 2005;352:1861-72.

[27] Shah RU, Freeman JV, Shilane D, et al: Procedural complications, rehospitalizations, and repeat procedures after catheter ablation for atrial fibrillation. *J Am Coll Cardiol* 2012;59(2):143-9.
